# Comparison of the efficacy of Sufentanil and Morphine Titration for patient-controlled Subcutaneous Analgesia in severe advanced cancer pain

**DOI:** 10.12669/pjms.39.2.6664

**Published:** 2023

**Authors:** Dong Liu, Wei Li, Li Chen

**Affiliations:** 1 Dong Liu, Department of Anesthesiology, Baoding No.1 Hospital, Baoding 071000, Hebei, China; 2 Wei Li, Department of Anesthesiology, Longyao county hospital, Xingtai 055350, Hebei, China; 3 Li Chen, Department of General Medicine, The Fourth Hospital of Hebei Medical University, Shijiazhuang 050011, Hebei, China

**Keywords:** Severe advanced cancer pain, Patient-controlled subcutaneous analgesia, titration, Morphine, Sufentanil

## Abstract

**Objective:**

To compare the efficacy and side effects of sufentanil and morphine titration for patient-controlled subcutaneous analgesia (PCSA) in severe advanced cancer pain management.

**Methods:**

A retrospective analysis was performed on the patients who were treated by two cancer centers with PCSA for severe advanced cancer pain at the Fourth Hospital of Hebei Medical University and Baoding No.1 Hospital between 2018 and 2021. These patients were divided into a sufentanil group and a morphine group. The drug dosage of the two groups was recorded. The pain intensity, sleep quality and adverse event rate (AER) were compared and analyzed between the two groups.

**Result:**

PCSA was successful in 95.2% (120/126) of the patients. In all cases, titration was successful within 24 hour, followed by oral administration of sustained-release opioid medications at 208.4 ±75.1 mg in the sufentanil group and at 207.9±66.3 mg in the morphine group. There was a significant difference in pain intensity and sleep quality before and after titration (P <0.05). Both groups exhibited a decline in their heart rates during titration. Compared with the baselines before titration, the mean heart rates were significantly reduced in both groups (P <0.05). The sufentanil group had an AER lower than that of the morphine group.

**Conclusion:**

Short-term use of sufentanil supports PCSA for patients with severe advanced cancer pain can achieve effective and rapid pain management, it is worth clinical implementation and application.

## INTRODUCTION

With the number of cancer patients increasing, cancer pain has become a major public health concern around the world.^1^ For moderate to severe cancer pain, opioid medications are accepted as the “gold standard”. In clinical practice, subcutaneous injection, intravenous injection, and patient-controlled subcutaneous analgesia (PCSA) represent the mainstay of rapid pain control.^2^-^4^ PCSA depends on subcutaneous drug delivery to achieve adequate pain control comparable to the intramuscular and intravenous routes and has advantages in complication rate, monitoring, management, nursing, patient compliance, safety, and medical costs. Therefore, it is ideal for implementation in the hospital or home care settings and is commonly used for pain and symptom control in palliative care.^5^

Morphine is a typical opioid drug usually used in the studies of other opioid medications as the control drug and is commonly used for rapid titration and maintenance therapy in advanced cancer pain management. However, morphine may sometimes produce strong adverse reactions, such as respiratory depression and hypotension.^6^,^7^ Compared with morphine, sufentanil is reported to take effect more quickly, have a lower AER and work more effectively to improve the quality of life of those undergoing advanced cancer pain treatment.^8^ This retrospective study was conducted to compare PCSA titration with sufentanil and morphine regarding their analgesic effects, safety profile and patient satisfaction levels by analyzing PCSA titration for severe advanced cancer pain management and switching to oral administration of opioid medications upon determination of target dosage via subcutaneous titration.

## METHODS

The retrospective analysis involved 126 patients who received PCSA for advanced cancer pain at the Fourth Hospital of Hebei Medical University and Baoding No.1 Hospital during 2018 and 2021. Patient data including demographic data, diagnosis of primary cancer, pre-PCSA use of opioid medications and baseline dosage were retrieved from electronic medical record systems. This study was approved by the Medical Ethics Committees of the Fourth Hospital of Hebei Medical University and Baoding No.1 Hospital on November 10, 2018 (No. [2018]108), and written informed consent was obtained from all participants or their family members.

### Inclusion criteria:

- Patients confirmed to have malignant solid tumors based on pathological or cytological diagnosis;
- Persistent, severe pain with a score of 7 and above at the onset based on the 11-point numeric rating scale (NRS), where ‘0’ represented ‘no pain’ and ‘10’ stood for ‘worst pain imaginable’.^6^,^9^,^10^
- All eligible patients were free of cognitive impairment and acted cooperatively in pain and satisfaction rating.

### Exclusion criteria:

- Patients who received PCSA for less than 24 hours or whose clinical data were meaningless;
- Titration failure due to any reasons.
- A total of 120 patients with successful titration were recruited for subsequent analysis, including 62 receiving PCSA titration with sufentanil and the rest 58 undergoing PCSA titration with morphine.

Continuous subcutaneous infusion (CSCI) was converted to oral opioid medications at the ratio of 1:2 - 2 mg of oral morphine was substituted for CSCI with 1mg of morphine. The potency ratio of morphine to sufentanil was 1:1000.^11^,^12^ *Titration method:* All forms of opioid medications were discontinued before titration to calculate the dose of opioid and convert to an equivalent dose of subcutaneous morphine or sufentanil (N0). *Dose determination dependent on pain intensity:* I. Mild (1 <NRS <4): increase by 25%; II. Moderate pain (4 ≤NRS <7): increase by 50%; III. Severe pain (NRS ≥7): increase by 75% to 100%. An estimated increase in the dose (N1) was determined. *Parameter setting:* The ratio of loading dose to background dose was 1.5. Background dose: (0.5-1) (N0+N1) / 24 h. PCA dose: (1-1.2) times the background dose. Lock-on: 15 minutes. Extreme limit: (1/4) (N0+N1)/h. The actual dose of subcutaneous titration for 24 hour (N2) was calculated and converted to the equivalent dose of controlled- or sustained-release opioid medications. Following the 24-hour titration, the PCA pump was used for another 12 hour and was not withdrawn until the controlled- or sustained-release opioid medications fully took effect.

(1) Pain intensity and sleep quality assessment: Pain intensity and sleep quality were evaluated before PCAS via titration using the NRS and the Pittsburgh Sleep Quality Index (PSQI), with the global PSQI score ranging from one to 21 and a higher score suggesting worse sleep quality. The following outcome measures were recorded at different time points (T0: before sufentanil or morphine titration; T1: 24 h after sufentanil or morphine titration; T2 and T3: 12 h and 24 h after administration of controlled- or sustained-release opioid medications): mean NRS score, blood pressure, heart rate, and PSQI score at different time points; adverse reactions such as respiratory depression, bradycardia, hypotension, drowsiness, nausea or vomiting, urinary retention, constipation, and itching.

### Statistical analysis:

The software SPSS19.0 was used for statistical analysis. Measurement data were expressed by “mean ± standard deviation (-χ±SD)”, examined by the paired sample t-test. Enumeration data were represented by percentage (%) and examined by the chi-squared (χ^2^) test, with the significance level at P <0.05.()

## RESULTS

**Fig.1:**
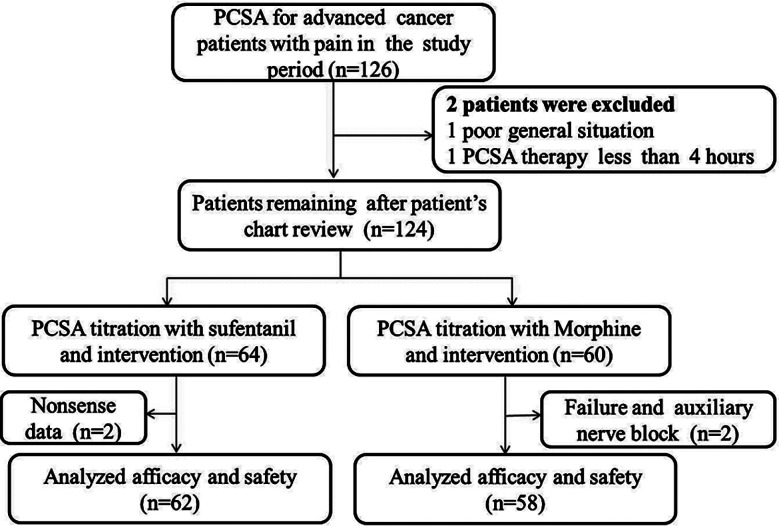
Study profile. *Abbreviation*; PCSA: patient controlled subcutaneous analgesia

A total of 126 patients receiving PCSA were included in this retrospective study. The study profile is shown in Fig.1. After screening, 120 patients with refractory cancer pain met the inclusion criteria, including 62 receiving PCSA titration with sufentanil and 58 being treated by PCSA titration with morphine. PCSA achieved pain relief in 95.2% (120/126) of the participants. Baseline characteristics of the eligible patients are given in Table-I.

**Table-I:** Patient characteristics.

Patients	Sufentanil group n(%)	Morphine group n(%)	P Value
Total, n	62	58	
Age (yrs)	64.9 ± 12.0	65.2 ± 11.6	0.894
Female/male, n	29/33	30/28	0.588
Weight (kg)	61.6±7.5	62.3±8.2	2.476
Pain intensity median (NRS)	8 (7-9)	8 (7-9)	
***Basic opioid use***			
Oxycodone sustained release tablets, n	38 (61.3%)	33 (56.9%)	0.625
Morphine sustained release tablets, n	19 (30.7%)	17 (29.3%)	0.873
Fentanyl transdermal patch, n	5 (8.1%)	8 (13.8%)	0.313
Equivalent oral morphine dose (mg)	147.4±67.0	144.8±55.5	0.819
***Pain type***			0.649
Nociceptive	42 (67.7%)	37 (63.8%)	
Nociceptive and neuropathic	20 (33.3%)	21 (36.2%)	
***Primary cancer location***			
Bronchopulmonary, n	18 (29.0%)	19 (32.8%)	0.659
Gastrointestinal, n	33 (53.2%)	30 (51.7%)	0.869
Head and neck, n	3 (4.8%)	2 (3.4%)	1.000
Urogenital, n	5 (8.1%)	4 (6.9%)	1.000
Others, n	3 (4.8%)	3 (5.2%)	1.000
Metastases present, n	53(85.5%)	51(87.9%)	0.694

### Efficacy:

**Figure.2:**
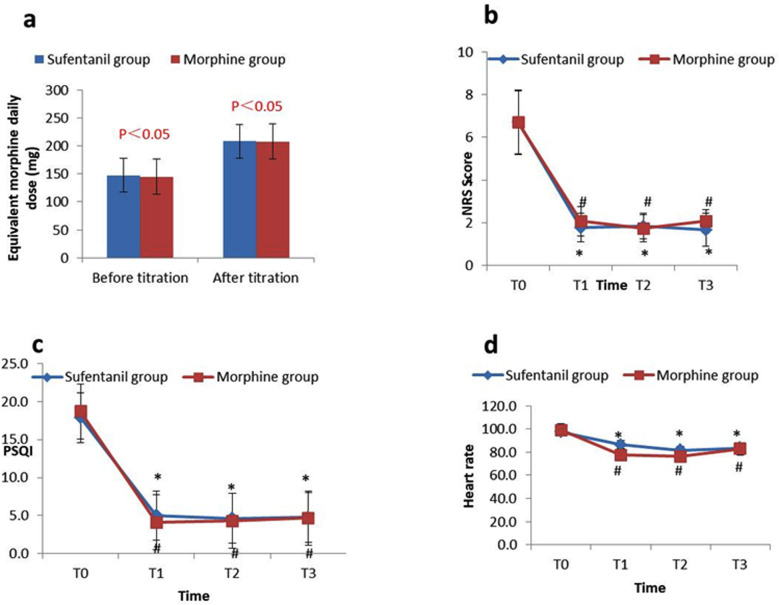
(a) The equivalent daily dose of morphine before and after titration in sufentanil group and morphine. (b) Changes of NRS scores before and after titration in sufentanil group and morphine group. (c) Changes of sleep quality in sufentanil group and morphine group before and after titration. (d) Changes of heart rate (beats/ min.) before and after titration in sufentanil group and morphine group. *P < 0.05, The comparison of sufentanil group before and after titration. #P < 0.05, The comparison of morphine group before and after titration.

In all cases, titration was successful within 24 hour. Opioid use did not differ greatly in either group before and after titration (P >0.05) (Fig.2a). The pre-titration NRS score in two groups suggesting a difference lacking statistical significance (P >0.05). The NRS scores at T1, T2, and T3 were significantly reduced as compared with the pre-titration NRS score (P <0.05, respectively), but the two groups displayed no statistically significant difference (P >0.05) (Fig.2b).

Both groups reported poor sleep quality before titration. The two groups exhibited a remarkable improvement in sleep quality at 24 hour after titration, which continued to improve after switching to the oral administration of controlled- or sustained-release opioid medications (Fig.2c), representing statistically significant differences as compared with the pre-titration PSQI scores (P < 0.05, respectively).

There was no significant difference in heart rate between the two groups before titration. After titration and switching to controlled- or sustained-release opioid medications, heart rates tended to decrease in both groups (P < 0.05, respectively), and yet the two groups showed no statistically significant difference (Fig.2d). Results of the statistical analysis showed that the AER of the sufentanil group was significantly lower than that of the morphine group (P < 0.05), see Table-II.

**Table-II:** Intergroup comparison of adverse events.

Adverse events	Sufentanil group n(%)	Morphine group n(%)	P-Value
***Severe adverse events (cases, percentage)***			
Hypoxemia (SPO2 <90%)	0 (0)	0 (0)	
Hypotension (SBP <90 mmHg)	1 (1.6%)	0 (0)	
Bradycardia (HR <45 bpm)	0 (0)	0 (0)	
Mild adverse events (cases, percentage)	11	39	0.000
Nausea or vomiting	2 (3.2%)	13 (22.4%)	
Dizziness	6 (9.7%)	14 (24.1%)	
Decrease of breath rate	2 (3.2%)	6 (10.3%)	
Constipation	0 (0)	3 (5.2%)	
Bradycardia (45 bpm <HR <60 bpm)	0 (0)	2 (3.4%)	
Hot flush and sweating	0 (0)	1 (1.7%)	
Hallucination	0 (0)	0 (0)	
Mild allergic reaction	0 (0)	0 (0)	
Catheter related or pump related adverse events	0 (0)	0 (0)	

## DISCUSSION

In China, about 4 million patients are newly diagnosed with cancer every year, and 60% to 70% experience cancer pain during the advanced stage.13,14 Clinicians mostly follow the WHO three step analgesic ladder to achieve satisfactory pain control by oral administration of controlled- or sustained-release opioid medications. For rapid relief of severe pain, clinicians should consider parenteral opioids via subcutaneous or intravenous injection. Titration is preferred to reduce side effects and ensure optimal pain control through dose adjustment. PCSA is suitable for patients with advanced cancer pain as it offers a convenient administration route in cancer pain management and nursing care, without seriously affecting daily activities or causing aeroembolism.15,16 PCSA is reported to yield a plateau concentration in 12-24 hour.5 In this study, mean NRS scores were remarkably reduced in both groups receiving PCSA titration with sufentanil or morphine, while higher patient satisfaction levels were observed; moreover, opioid-related adverse reactions were not evident, and catheter- or pump-related adverse events did not occur throughout the treatment course.

Injectable morphine is currently the clinical mainstay of titration or maintenance therapy for advanced cancer pain management. Morphine binds to glucuronide to produce morphine-3-glucuronide (M3G) and morphine-6-glucuronide (M6G). As a highly hydrophilic metabolite, M6G displays strong analgesic properties; in contrast, the other metabolite M3G appears to be associated with morphine-related neurotoxicity, which cannot bind to opioid receptors or stimulate analgesic activities.17 Additionally, injectable morphine can induce severe adverse reactions such as respiratory depression, dizziness, nausea, vomiting. In this study, 58 patients were treated by PCSA titration with morphine and 62 by PCSA titration with sufentanil. PCSA titration was successful in all cases within 24 h and then switched to sustained-release oral opioid medications. This demonstrated the feasibility and practicability of short-term PCSA titration with sufentanil or morphine to achieve satisfactory analgesia, where adverse reactions occurred less frequently in the sufentanil group as compared with the morphine group.

Sufentaniln is a 4-Anilinopiperidine derivative like fentanyl. The opioid analgesic sufentanil is one thousand times more potent than morphine and surpasses morphine in safety and analgesic threshold.11 Pharmacokinetics demonstrates that sufentanil is especially effective in treating cancer patients with renal impairment.18 Sufentanil is reported to work faster in cancer pain management, where patients are less likely to develop opioid resistance.19 The statistical analysis in this study showed that sufentanil titration did not increase opioid use or induce drug accumulation or poisoning by 24-hour rapid titration. In short, sufentanil is comparable to morphine in PCSA titration for rapid pain control. Meanwhile, adverse events occurred less frequently in PCSA titration with sufentanil as compared with PCSA titration with morphine, conforming to the findings reported by Wan CF et al.8 Intense pain is known to trigger physical stress that induces increased pulse rate, blood pressure and respiratory rate. The study results demonstrated reduced heart rates in both groups after titration probably because of effective pain control.

### Limitations of the study:

This retrospective study has its own limitations as no data is available to investigate the effects of PCSA on anxiety, depression and quality of life in cancer patients. In addition, this study did not cover the difference in hospital costs between rapid titration with wireless analgesic pump and conventional titration.

## CONCLUSIONS

Short-term use of sufentanil supports PCSA for patients with severe advanced cancer pain to achieve effective and rapid pain management with a relatively low AER and can be successfully switched to controlled- or sustained-release opioid medications.

### Authors’ Contributions:

**DL** designed this study. prepared this manuscript, are responsible and accountable for the accuracy and integrity of the work.

**LC** collected and analyzed clinical data.

**WL** Data analysis, significantly revised this manuscript.
